# Bone phenotypes in rheumatology – there is more to bone than just bone

**DOI:** 10.1186/s12891-020-03804-2

**Published:** 2020-11-28

**Authors:** Christian S. Thudium, Signe Holm Nielsen, Samra Sardar, Ali Mobasheri, Willem Evert van Spil, Rik Lories, Kim Henriksen, Anne-Christine Bay-Jensen, Morten A. Karsdal

**Affiliations:** 1 Nordic Bioscience, Herlev Hovedgade, 205-207 Herlev, Denmark; 2 Biotechnology and Biomedicine, Technical University of Denmark, Lyngby, Denmark; 3 Research Unit of Medical Imaging, Physics and Technology, Faculty of Medicine, University of Oulu, Oulu, Finland; 4 Department of Regenerative Medicine, State Research Institute Centre for Innovative Medicine, Vilnius, Lithuania; 5 Department of Orthopedics, University Medical Center Utrecht, Utrecht, The Netherlands; 6 Centre for Sport, Exercise and Osteoarthritis Versus Arthritis, Queen’s, Medical Centre, Nottingham, UK; 7 Department of Rheumatology and Clinical Immunology, University Medical Center Utrecht, Utrecht, the Netherlands; 8 Department Of Rheumatology, Dijklander Hospital, Hoorn, The Netherlands; 9 Skeletal Biology and Engineering Research Center, Laboratory of Tissue Homeostasis and Disease, KU Leuven, Leuven, Belgium

**Keywords:** Osteoarthritis, Psoriatic arthritis, Rheumatoid arthritis, Ankylosing spondylitis, Endotype, Matrix, Biomarker, Biochemical marker, Phenotype, Bone, Remodeling, Endochondral, Therapeutic

## Abstract

Osteoarthritis, rheumatoid arthritis, psoriatic arthritis, and ankylosing spondylitis, all have one clear common denominator; an altered turnover of bone. However, this may be more complex than a simple change in bone matrix and mineral turnover. While these diseases share a common tissue axis, their manifestations in the area of pathology are highly diverse, ranging from sclerosis to erosion of bone in different regions. The management of these diseases will benefit from a deeper understanding of the local versus systemic effects, the relation to the equilibrium of the bone balance (i.e., bone formation versus bone resorption), and the physiological and pathophysiological phenotypes of the cells involved (e.g., osteoblasts, osteoclasts, osteocytes and chondrocytes). For example, the process of endochondral bone formation in chondrocytes occurs exists during skeletal development and healthy conditions, but also in pathological conditions. This review focuses on the complex molecular and cellular taxonomy of bone in the context of rheumatological diseases that alter bone matrix composition and maintenance, giving rise to different bone turnover phenotypes, and how biomarkers (biochemical markers) can be applied to potentially describe specific bone phenotypic tissue profiles.

## Background

**Fig. 1:**
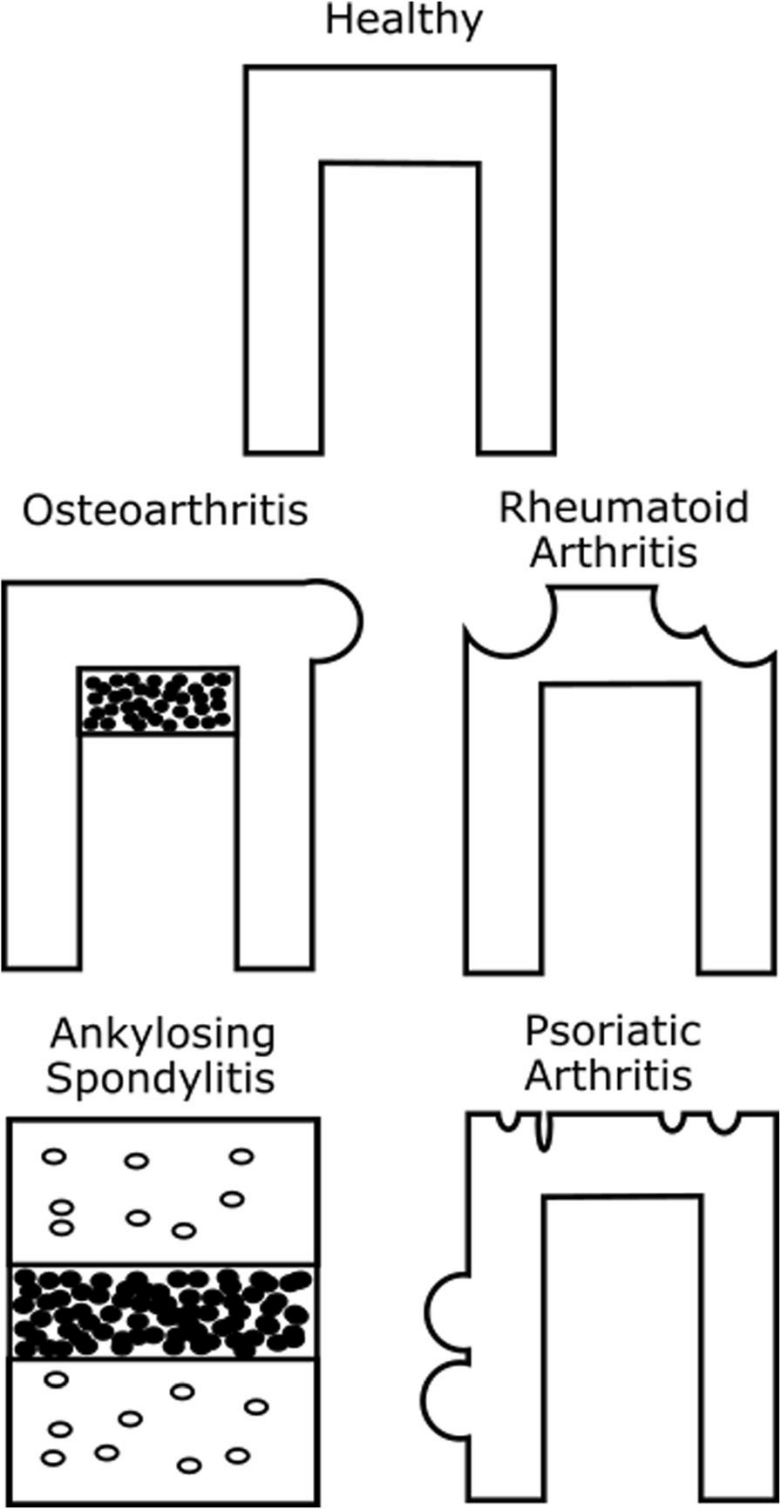
Schematic representation of epiphysial and vertebral bone phenotypes in musculoskeletal and rheumatic diseases. OA is characterized by increase bone remodeling and progressive subchondral thinning followed by sclerostation. Mechanical adaptation may lead to the formation of osteophytes at the joint margins. The bone phenotype in RA joints are usually showing as excessive bone erosions. Hallmarks of AS consists of ankylosis of the spine, but also pathological bone loss both systemically and locally. The bone phenotype in psoriatic arthritis is mixed but characterized by bone erosions similar to RA. These are often less severe and more asymmetric in terms of affected joints. PsA also presents with pathological bone formation in the axial skeleton such as syndesmophytes, and in the peripheral joints, as joint ankylosis, enthesophytes or periosteal bone formation

Maintenance of bone homeostasis is essential for proper function of the skeleton. Mechanical stress, trauma, autoimmunity, ageing, menopause and other hormonal changes, genetics, diet and lifestyle lead to alterations in bone and joint structures and can result in pathological changes. Aberrant bone turnover is a feature shared by many rheumatic diseases, including osteoarthritis (OA) (although strictly speaking not a rheumatic disease), rheumatoid arthritis (RA), psoriatic arthritis (PsA), and ankylosing spondylitis (AS) [1–4]. The bone alterations in these diseases are manifested in different forms (Fig. 1). Increased remodeling and sclerosis, bone marrow lesions, and increased vascularization in the subchondral bone and osteophytes [5] are all characteristic hallmarks of OA, with changes to the bone occurring slowly over many years. In RA, typical bone changes are bone erosions at the joint margins and periarticular and systemic bone loss due to the chronic and high-grade inflammatory state of the disease [6, 7]. PsA bone changes consist of localized bone erosions in the articular area as well, but in contrast to RA, PsA also involves characteristic ossification of entheses [8]. The main features of AS are ankylosis of the sacroiliac joints and the spine resulting in limited range of movement and increased fracture risk [1]. A large body of research has focused on the molecular basis of the bone changes in these diseases and this work has increased our understanding of how cellular function and matrix changes affect the manifestations of disease pathology. While these diseases are quite different in their etiology, molecular origin and pathology, many of the bone manifestations share similarities such as increased remodeling, and endochondral ossification-like changes in subgroups of patients. This suggests that there may be opportunities for targeting new therapeutics against specific endotypes within and across disease indications given that these can be identified. However, in order to do so, a solid characterization and deep understanding of pathological bone changes both in the individual patient and within diseases in general is needed.

The aim of this review is to review the relevant literature in order to provide an understanding of the bone manifestations underlying joint diseases in rheumatology, and how these may be characterized using biomarkers to identify potential bone specific endotypes within and across rheumatic diseases.

### What is endochondral bone formation?

Endochondral ossification, or endochondral bone formation, is the process underlying longitudinal bone growth during skeletal maturation. A significant reason for the interest in this process is the close similarity between endochondral bone formation and the bone pathology of for example the SpA joint [5, 9–11]. In healthy individuals the process is initiated when mesenchymal cells proliferate and differentiate into prechondroblasts and further into chondroblasts. The chondroblasts become embedded in the cartilaginous matrix they secrete, and further differentiate into chondrocytes forming a very early bone rudiment. The chondrocytes mainly produce matrix molecules, but also secrete growth factors, such as vascular epithelial growth factor (VEGF) and receptor activator of nuclear factor kappa-Β ligand (RANKL) that stimulate vascular invasion and recruitment of osteoclasts [12]. The rudiment is surrounded by differentiating mesenchymal cells developing into the periosteum, and osteoblasts start to lay down the osteoid layer [13, 14].

Mononuclear osteoclast precursors and endothelial cells invade the osteoid resulting in capillary invasion and formation of bone marrow cavity. Chondrocytes in the growth plate proliferate in columns towards the diaphysis, becoming hypertrophied as they deposit cartilage matrix and finally undergoing apoptosis [15]. Longitudinal growth results from the continued proliferation of the chondrocytes leading to calcification of the matrix in the growth plates, followed by the resorption, formation and vascularization by osteoclasts, osteoblasts and endothelial cells respectively. These subsequent steps lead to a gradual diminishing and finally the disappearance of the growth plates. The result is an arrest in longitudinal growth [16].

Inflammatory signaling such as tumor necrosis factor α (TNFα) and interleukin 23 (IL-23) or altered expression of growth factors such as bone morphogenic proteins (BMPs) and Wnts may disturb or misdirect these processes depending anatomical site and/or external stimuli such as mechanical changes as seen in various arthritic diseases including RA and SpA [1, 7].

### What is normal bone remodeling?

Bone remodeling is required for maintenance of calcium homeostasis and strength of the bones and thus for the sustained health and functionality of the skeleton [17]. Bone remodeling involves the combined functions of both osteoclasts, osteoblasts and osteocytes and tightly regulated and stepwise process leading to replenishment of the skeleton throughout life [2, 18].

In healthy adults bone resorption is always followed by a balanced amount of bone formation in a process termed coupling [19]. Coupling has since the 1960 been described as a targeted and balanced induction of bone formation by osteoblasts in response to bone resorption [18, 20–22]. Imbalances in coupling and bone remodeling caused by either hormonal changes or genetic defects underlie pathological conditions in primary bone diseases, such as osteoporosis (OP) or osteopetrosis (OPT), but are also part of the disease process in OA, RA and other rheumatic diseases [2, 11]. The impact of inflammation on bone have been shown to be dependent on the site affected, cell types, mechanical environment and ultimately the cytokines and molecular factors present in the microenvironment. Inflammatory signaling mediated mainly by cytokines are central to uncoupling of osteoclast and osteoblast function leading to inflammation induced bone loss and pathological bone formation in a disruption of normal bone remodeling [23].

### Lessons learned from other bone diseases

Pathologies arising from alterations in how bone is formed and resorbed are numerous, and include different forms of osteoporosis, calcium metabolic disorders and severe genetic malignancies; Such diseases have been instrumental in understanding bone processes, cellular communication of the bone, and identifying potential markers describing bone compartment changes. Here, we have selected three non-rheumatic situations underlining extremes of osteoclast and osteoblast function and bone modeling and remodeling gone wrong, namely hypogonadal (or postmenopausal osteoporosis), osteopetrosis and fibrodysplasia ossificans progressive (FOP).

### Osteoporosis – the systemic bone loss phenotype

Hypogonadal osteoporosis arises from a decrease of sex steroid production [24]. As sex steroids directly reduce osteoclastogenesis, this reduction in the sex steroid levels leads to an increase in osteoclastogenesis and thereby osteoclast numbers and subsequently increased bone resorption [25]. As a function of the coupling between bone resorption and bone formation, bone formation is also increased. However, the increase in bone resorption is not completely countered by the increase in bone formation, resulting in net bone loss. Ultimately, this results in lower bone mass, deterioration of the microarchitecture of bone leading to fragility and an increased risk of fractures [26].

Most therapies for osteoporosis target the accelerated osteoclastogenesis and/or function. Bisphosphonates, denosumab (anti-RANKL antibody), hormone replacement therapy and Selective Estrogen Receptor Modulators (SERMs) focus on reducing the number of osteoclasts and, thereby, bone resorption [27]. Due to the coupled nature of the bone remodeling process, these therapeutic treatments are associated with secondary decreases in bone formation [19, 25]. Biomarkers have been extensively applied in osteoporosis clinical research, and markers such as the bone resorption marker C-terminal telopeptide I (CTX-I) and the bone formation markers amino-terminal propeptide of type 1 procollagen (PINP) and osteocalcin are measured routinely in most clinical studies and pharmacological trials (Table 1). While the level of CTX-I in the individual patient says very little about the state of the bone in the individual patient, the dynamic quantification, the ratio with the bone formation marker osteocalcin, and the response to for example anti-resorptive agents provides important information about tissue turnover rates and prognosis, information which is difficult to obtain by other standard methods such as x-rays. Such information have enabled rapid decision making in clinical osteoporosis trials [36].

**Table 1:** Biomarkers of bone and cartilage turnover in osteoporosis, osteopetrosis and fibrodysplasia ossificans progressive

Biomarker	Disease levels compared to healthy	Findings	References
**Bone turnover**			
CTX-I	Osteoporosis: not diagnostic Osteopetrosis: increased in in vivo models	Osteoporosis: Reduced by anti-resorptive agents (eg. bisphosphonates and anti-RANKL	[28, 29]
PINP	Osteoporosis: not diagnostic	Osteoporosis: Modulated in response to pharmacological interventions such as bisphosphonates, anti-SOST and anti-RANKL.	[28, 29]
Osteocalcin	Osteoporosis: not diagnostic Osteopetrosis: reduced in ADOII patients FOP: inconclusive	Osteoporosis: Modulated in response to pharmacological interventions such as bisphosphonates, anti-sclerostin and anti-RANKL. FOP: Age dependent association with mortality	[28–30]
(B)ALP	Osteoporosis: not diagnostic Osteopetrosis: no difference in ADOII patients	Osteoporosis: Modulated in response to pharmacological interventions such as bisphosphonates, anti-sclerostin and anti-RANKL.	[28, 29, 31]
TRACP5b	Osteoporosis: Elevated compared to healthy controls Osteopetrosis: Increased in ADOII Increased in in vivo models	Osteoporosis: associated with markers of bone remodeling and BMD. Osteopetrosis: associated with increased fractures in ADOII patients	[32–35]

*ADOII* Autosomal dominant osteopetrosis, *SOST* Sclerostin, *BMD* Bone mineral density

### Osteopetrosis – the systemic bone gain phenotype

OPT is a rare, inherited high bone mass disease [37]. The bones are in general dense, yet frail, and patients present with multiple fractures. In severe forms the lack of a bone marrow cavity results in extramedullary hematopoiesis in the liver and spleen, which, if left untreated, can fall short and lead to anemia and death [38, 39]. In general, two types of OPT exist. One type is osteoclast-poor and is caused by molecular defects in osteoclastogenesis, such as the RANKL-RANK system [40–43]. The other type is osteoclast-rich and caused by defects in genes involved in bone resorption and presents with numerous non-functional osteoclasts [37, 38, 44].

An interesting difference between the two types of OPT lies within the overall bone turnover. Both types show reduced bone resorption and the osteoclast-poor type shows reduced bone formation as expected from the coupling between these processes [40]. Importantly though, the osteoclast-rich type shows normal or increased bone formation rates, indicating that the presence of osteoclasts, but not their resorption, is essential for bone formation [32]. Biomarkers have been a valuable tool in characterizing the discrepancies between the different forms of ostepetrosis by allowing quantification of osteoclast numbers (such as tartrate-resistant acid phosphatase (TRACP) 5b), the bone resorption (CTX-I and collagen type I C-terminal telopeptide (ICTP)) and bone formation rates (alkaline phosphatase (ALP) and osteocalcin) and thereby essentially interrogating their respective functions both clinically and pre-clinically (Table 1) [31–34, 45].

### Fibrodysplasia ossificans progressiva – the systemic ossification phenotype

FOP is a rare genetic disease characterized by abnormal heterotopic ossification of the muscles, tendons, aponeurons, and ligaments. With progressive ossification affecting peri-articular and soft tissues the disease will lead to skeletal deformities growth impairment, joint ankylosis and chronic pain affecting gait, posture and respiration. The median life expectancy of individuals with FOP is approximately 40 years [46]. The molecular mechanism of disease is well documented and includes mutations in the Activin A receptor type I (ACVR1) gene encoding for the activin-like kinase 2 (ALK2) [47], a BMP type I receptor. BMPs regulate a diverse range of cellular activities such as stem-cell renewal, differentiation, proliferation, migration and apoptosis and also include the induction of the pathway of endochondral bone [48]. These mutations seem to make mesenchymal progenitors more prone to chondrogenic and osteogenic differentiation through increased or constitutive BMP/Smad reporter [49–52]. These findings highlight the importance and delicate balance in skeletal tissue growth and showcase in the extreme circumstance the devastating effects of unchecked endochondral ossification. Only few biomarker studies have been undertaken in FOP patients, likely due to the detrimental effects of even light invasive techniques such as blood sampling (Table 1). The only report on bone markers in serum, to our knowledge, include bone formation markers osteocalcin and ALP but focus has been on aspects such as mortality rather than structural alterations, making it difficult to interpret pathology derived alterations in levels [30].

### Osteoarthritis – the mechano-adaptive phenotype

OA is the most common form of chronic arthritis [53, 54]. A hallmark of the disease is progressive degeneration of articular cartilage. A strong body of evidence from both animal and human observations suggests that the structural integrity of articular cartilage, besides normal chondrocyte function, is associated with a physiological subchondral bone turnover and mechanical stress [55, 56]. It now appears to become evident that subchondral bone turnover and its interaction with the articular cartilage are important for joint homeostasis [57–60]. Articular chondrocytes and cells in the subchondral bone register molecular stress signals, load and strain and modulate the bone-cartilage mechanical unit accordingly. OA cartilage is characterized by insufficient homeostatic and reparative processes, unable to compensate for the destructive mechanisms leading to deterioration of joint structure. The main bone related features include increased subchondral remodeling, thickening of the subchondral bone plate, osteophytes, and bone marrow lesions. The thickening of the subchondral cortical bone plate and increased bone turnover are likely to be a consequence of OA, but may, in turn, also further contribute to the altered composition and biomechanical properties of the OA joint [61–65]. More recent preclinical studies have been crucial in providing information on the early role of bone turnover in disease progression [57–60].

**Fig. 2:**
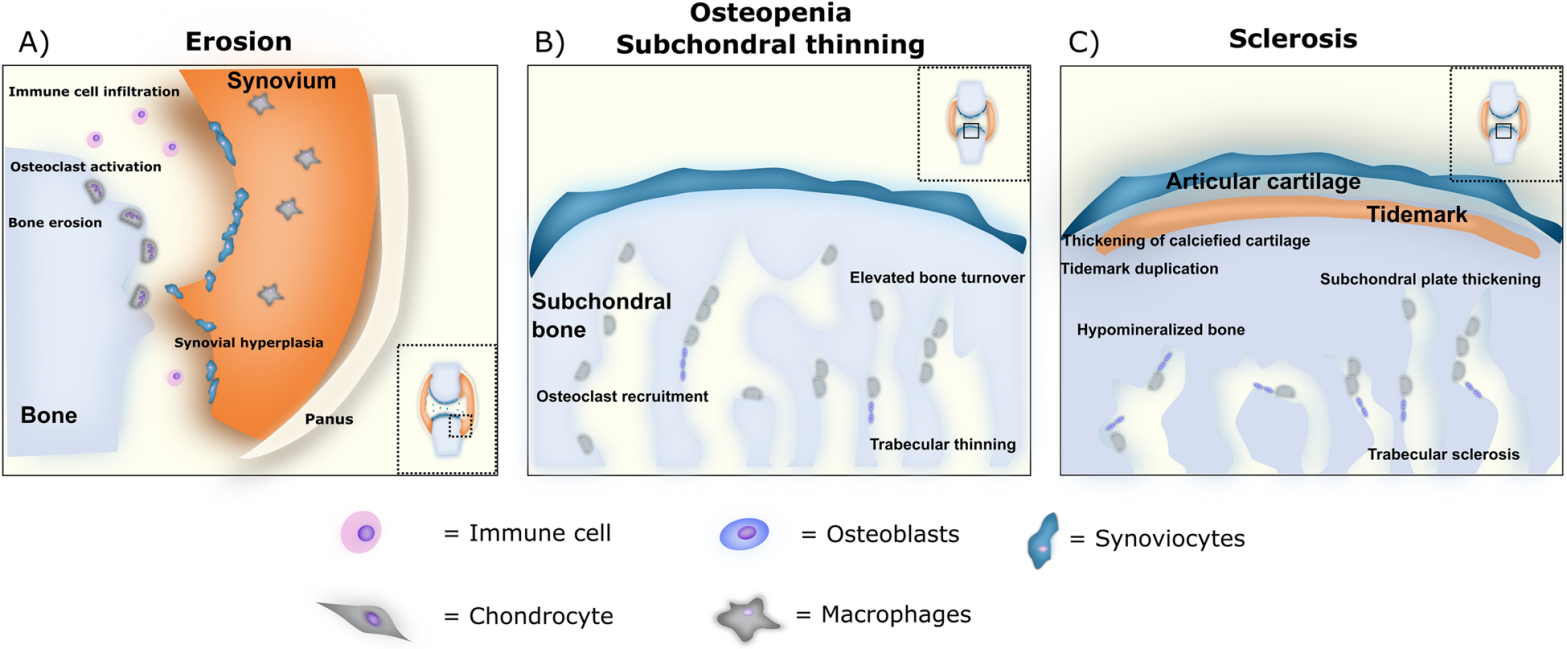
Pathological bone remodeling phenotypes (**a**) Synovial inflammation, pannus formation and immune cell infiltration is associated with increased release of osteoclastogenic cytokines which drives osteoclast recruitment and differentiation resulting in aberrant bone erosive processes. **b** In OA increased subchondral remodeling is lead inflammatory changes or mechanical alterations cause infiltration of bone cells from the marrow and vascularization into the subchondral bone area leading to increased bone remodeling and instances of subchondral thinning. In RA, osteopenia localizes in the periarticular regions. A combination of increased cytokine signaling and inflammation from the bone marrow may activate osteoclastogenesis. At the same time osteoblast mediated bone formation is inhibited by anti-osteogenic factors such as DKK1 and SOST. **c** In OA, bone sclerosis occurs in response to increased mechanical loading, resulting in excessive bone formation, thickening of the subchondral bone plate and calcified cartilage, tidemark duplication and reduced mineralization of the bone

Studies of the periarticular bone in knee and hip OA patients show abnormal subchondral bone, with sclerosis, thinning of the vertical trabecular structure, and under-mineralization, thought to be result from accelerated bone remodeling (Fig. 2) [66, 67]. Excessive bone remodeling was first exemplified by bone scintigraphy which strongly indicated the importance of subchondral bone remodeling as a characteristic feature of OA [68–70]. The state of mineralization is profoundly affected by the rate of bone remodeling, high bone remodeling rates attenuating the late phase of mineral deposition leading to a hypomineralized state and a reduction in the elastic modulus [71]. This suggests that the subchondral bone in OA is adapting to altered mechanical load or damage, perhaps as a form of stress-shielding [72]. Repetitive excessive loading may result in damage at local sites resulting in a form of microdamage associated with microcracks at which local target remodeling may occur [73]. This type of remodeling is hypothesized to, at least partly, be related to the formation of bone marrow lesions (BMLs), as can be visualized by magnetic resonance imaging (MRI) in OA patients [73]. Histological studies of BMLs from hip OA patients have revealed the presence of microcracks, different stages of healing, fibrous tissue deposition and increased bone remodeling, all indicating a reparative bone remodeling process [73] and an association between mechanical and traumatic etiologies and subchondral bone marrow changes. In relation to structure, studies have also shown that BML size correlates with cartilage damage and predicts cartilage loss longitudinally [74]. In line with this, microarray studies of BML tissue isolates have shown increased expression of proteases, osteogenic and chondrogenic molecules such as matrix metalloproteinase (MMP) 13 and molecules associated with Wnt signaling, suggestive of local repair processes and heavy remodeling [75].

OA development is associated with changes in chondrocyte behavior, including matrix calcification and expression of hypertrophic markers such as MMP13 and type X collagen, which are both signs of hypertrophy [76, 77]. These phenomena, in many cases, appear similar to the function of hypertrophic chondrocytes in the growth plate of long bones undergoing endochondral ossification during growth. While the hypertrophic phenotype in development is a key step in the calcification of new bones, hypertrophy in OA may not only be characterized by increased calcification. Hypertrophy also contributes to a pro-inflammatory environment in the cartilage, with high expression of matrix degradation enzymes causing the hypertrophic chondrocytes to actively degrade their surroundings [78, 79]. In addition, the matrix associated with these differentiated chondrocytes may be biomechanically inferior for the bone-cartilage unit and further contribute to progression of osteoarthritis.

**Fig. 3:**
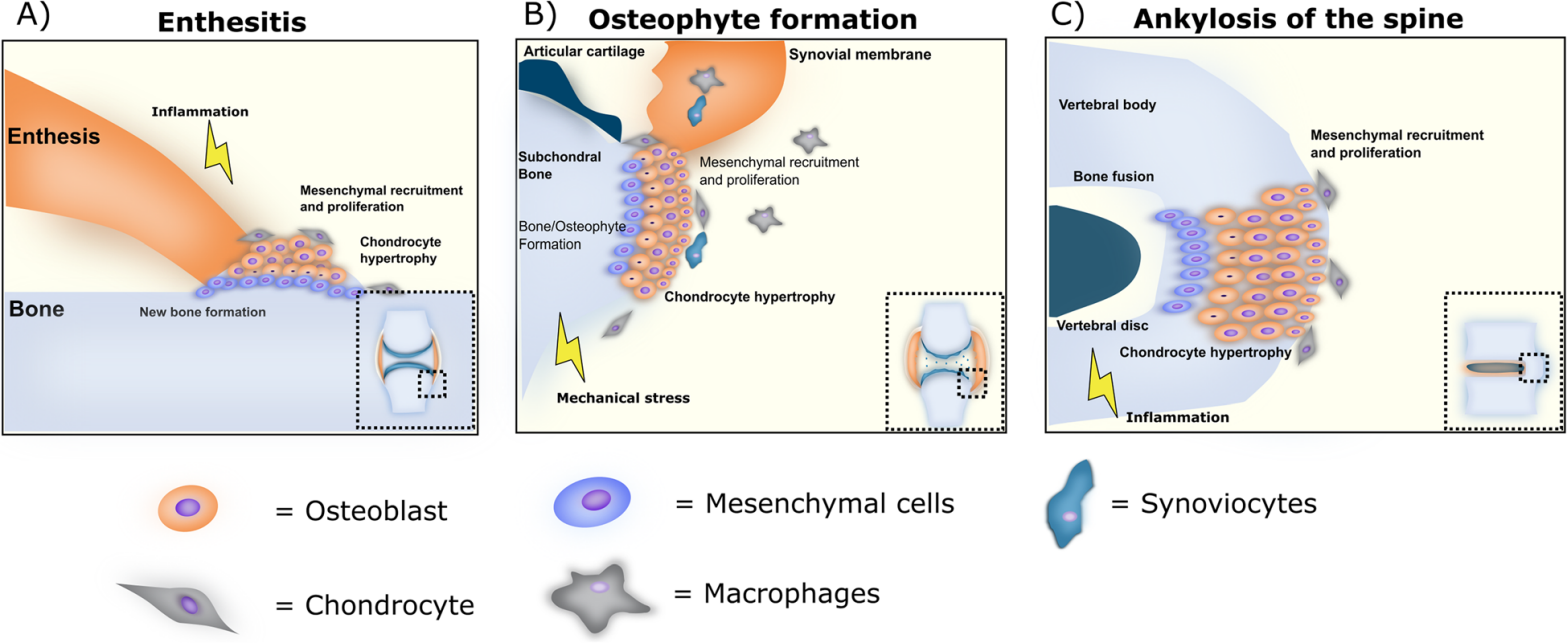
Proposed phenotypes of endochondral bone formation in rheumatic diseases (**a**) Mechanical or inflammatory signals may initiate mesenchymal recruitment chondrocyte hypertrophy and osteoblast mediated bone formation in a stepwise process mimicking endochondral bone formation. **b** Similarly, excessive mechanical stress may lead to activation of chondrocytes at the periarticular bone, leading to increased hypertrophy, osteoblast recruitment from the vasculature and endochondral bone formation. **c** Mechanical stress, inflammation and genetic predisposition, may cause alteration in cytokine expression, including BMP and Wnt signaling leading to mesenchymal recruitment and proliferation followed by endochondral bone formation leading to ankylosis of the spine

While not occuring in the subcondral region one of the cardinal bone alterations in OA is osteophytes (Fig. 3). These bony outgrowths occur at the joint margins adding new bone by endochondral ossification normally associated with development and skeletal growth. The biomechanical function of osteophytes is not clear but they may serve to stabilize the joint and affect joint mobility. The local production of growth factors has been implicated to orchestrate this process, including transforming growht factor (TGF) β and BMP2 [80–82].

In summary, changes in the subchondral region are associated with bone marrow changes, BMLs, microfractures and vascularization, leading to significant increases in bone remodeling and tissue turnover causing perturbed bone structure in the joint [9]. Not surprisingly it has been proposed that novel pharmacological treatments for OA should include treatments targeting the subchondral bone alterations. Biomarkers that can accurately characterize and identify patients with a bone driven endotype would be useful in both drug development and later personalized medicine. When discussing endotypes, such markers may also be useful in drug repositioning; adapting drugs from - or targeting similar endotypes in other joint diseases such as PsA and AS, which share similar features such as calcification of the interface between bone and other tissues.

### Tissue turnover markers in osteoarthritis

The heterogenous nature of OA pathology has driven the field towards defining OA phenotypes and developing and applying biomarkers to describe disease state and to predict prognosis and treatment response. The extracellular matrix (ECM) centralized pathology of OA has centered efforts around proteins and peptides arising from the remodeling of the different joint matrices, including bone and cartilage (Table 2). Here the cleavage products from the proteolytic burden associated with OA pathology have proven useful as biomarker targets for describing disease pathology. Despite successes, their utility in OA has still been somewhat limited compared to RA, among others, which is most likely due to the relatively slow disease course and a large systemic background arising from the local nature of OA [105]. Associated with the degradation of cartilage the MMP-derived CTX-II is the most well-described urinary marker in OA. In clinical studies CTX-II has been shown to correlate with Kellgren-Lawrence (KL) grade in OA patients [87], bone marrow lesions [88], osteophytes [89] and with the incidence and progression of radiographic OA [89–92]. C2M, measured in serum, another MMP generated fragment, is also associated with cartilage degradation and found to be higher in knee OA patients compared to healthy controls [106]; as are serum Coll2–1 and C2C [96]. Type X collagen is a well-known marker of chondrocyte hypertrophy [79]. A recent study in knee OA patients identifies a neo-epitope fragment of type X collagen generated by Cathepsin K, Col10neo. In this study, the fragment was localized to the pericellular matrix of chondrocytes and at sites of cartilage fibrillation in line with increased chondrocyte expression and local proteolytic activity. The marker was also associated with increased severity defined by KL grade. Interestingly the level of Col10neo was higher in OA patients compared to RA patients suggesting that type X collagen expression and chondrocyte hypertrophy not surprisingly is more pronounced in OA compared to RA [76, 107].

**Table 2:** Biomarkers at the bone cartilage interface in OA

Biomarker	Disease levels compared to healthy	Findings	References
**Bone turnover**			
CTX-I	–	Increased in endotype subpopulation	[83]
αCTX	–	Associated with increased bone turnover, and progression of disease (JSN, osteophytes) in OA patients	[84]
C1M	Increased	Increased in total joint replacement subpopulation	[85]
TRACP	–	Associated with subchondral osteoclast number and pain in symptomatic knee OA	[86]
**Cartilage turnover**			
CTX-II	Increased	Correlated with KL grade, bone marrow lesions, osteophytes. Associated with incidence and radiographic progression of OA	[87–94]
C2M	Increased	Associated with KL grade	[95]
Coll2–1	Increased	Proposed association with progression of OA	[96]
C2C	Increased	–	
PIIANP	–	Associated with progression of knee OA. Negatively associated with disease burden in knee and hip OA	[97–99]
PIIBNP	Decreased	–	[100]
ARGS	Increased	–	[101]
COMP	–	Associated with incidence and progression of OA	[102–104]

*JSN* Joint space narrowing

Although not a neo-epitope, the cartilage residing cartilage oligomeric protein (COMP) is increased in serum and synovial fluid of OA patients [102, 103], and one study has shown that high baseline levels predict development of radiograph knee OA [104].

The successful application of biomarkers in clinical trials in the osteoporosis field has further ensured access to already validated markers such as urinary and serum CTX-I [108] and its non-isomerized counterpart αCTX (measured in urin) [109, 110]. With the acknowledgement of the importance of bone in OA pathology, measuring bone-related biomarkers in OA has gained traction. αCTX has been associated with increased bone turnover measured by bone scintigraphy and with progression of disease based on joint space narrowing and osteophyte score in symptomatic radiographic knee OA patients [84]. In patients with symptomatic knee OA, serum TRACP5b, an enzyme secreted by osteoclasts has been associated with the number of osteoclasts in the subchondral area. Furthermore, these levels were associated with current pain and pain change, suggesting osteoclast involvement in the subchondral bone area in the development of symptomatic OA [86]. While formation markers in cartilage such as amino-terminal propeptide of type 1 procollagen A and B (PIIANP and PIIBNP) have shown associations with chondrocyte activity and anabolic actions [97, 100, 111], bone formation markers such as serum PINP and osteocalcin have failed to show consistent associations with OA pathology.

While an established definition of endotypes is still lacking in OA, studies are beginning to utilize biomarkers to explore and better understand the underlying changes in OA and how to separate these into categories that can be used to optimize treatment [112–114]. Future work should include clear definition of endotypes through careful characterization of both the molecular pathology as well as the structural changes occurring over time as well as with intervention. For this, well characterized longitudinal cohorts are needed to investigate the changes in biomarkers over time in different subgroups. Well executed clinical trials testing efficacious disease modifying OA drugs (DMOADS) will also be crucial to understand the changes in biomarkers and how this affects potential endotype definitions.

### Rheumatoid arthritis – the bone erosion phenotype

RA is a complex autoimmune inflammatory disease that primarily affects peripheral joints, presenting with or without systemic, extra-articular involvement. RA is histologically characterized by synovial inflammation (synovitis) and hyperplasia leading to the formation of invasive pannus tissue. The pannus invades and erodes the adjacent cartilage and bone tissue leading to cartilage destruction and bone loss, characteristic of RA (Fig. 2) [115, 116]. Both trabecular and cortical types of bone are part of the bone loss in RA and three patterns of bone loss- focal, juxta-articular and systemic have been described [6, 117]. Though governed by different cellular and pathological mechanisms, they share common pathologic bone remodeling, whereby bone resorption by osteoclasts is increased and bone formation by osteoblasts is impaired. This uncoupling of bone resorption and bone formation leads to net osteoporosis [118].

Focal bone loss in RA appears in the form of bone erosions and subchondral osteolysis at the articular surface. Several lines of evidence indicate that the erosive process is carried out by osteoclasts that populate the interface between pannus and articular bone [6, 119–122], and is driven in part by cytokines with osteoclastogenic potential, produced by the RA synovium (Fig. 2). The synovial infiltrate is a rich source of RANKL, macrophage colony-stimulating factor (M-CSF), IL-1, IL-17, and TNFα, all of which can drive the development of osteoclasts from their macrophage precursors [123], as described in the previous section. The main sources of these cytokines are macrophages and fibroblast-like synoviocytes (FLS), and the number and activation of macrophages at the bone-pannus interface significantly correlate with the degree of the bone damage [93]. T- and B- lymphocytes in the inflamed synovium also contribute to the inflammation-induced bone loss by modulating the RANKL/osteoprotegerin (OPG) system through release of pro-inflammatory cytokines such as IL-6, TNFα and IL-17A [116, 124]. Additionally, mature B cells and T cells can directly promote bone loss by producing RANKL, one of the key cytokines controlling osteoclast differentiation [125], and by B-cell mediated autoantibody production, as discussed below.

The second form of focal bone loss characterizing RA is periarticular osteopenia, the loss of trabecular size and number in the metaphyseal regions adjacent to the inflamed joints. Periarticular osteopenia is also believed to be mediated by osteoclasts, albeit through different mechanisms [117]. As periarticular bone is not directly in contact with pannus, the osteopenia is most likely an effect of immune cells in the bone marrow compartment and circulating cytokines. Of importance, periarticular bone loss seen in RA is not only due to increased production and activity, and possibly decreased apoptosis of osteoclasts [123, 126], but also due to impaired bone repair by osteoblasts [118]. This virtual absence of bone-formation (repair) response in RA bone, in comparison to other types of inflammatory arthritis, suggests the involvement of signals that block new bone formation. Studies on human and animal tissues have provided compelling evidence that increased levels of antagonists of the Wnt signaling pathway like Dickkopf-related protein 1 (DKK1), soluble Frizzled-related protein 1 (sFRP1) and sclerostin (SOST) in the inflammatory milieu of RA synovium might be responsible for the phenotype [6, 127]. TNFα - the major inflammatory cytokine in RA – has been suggested as an ideal link between the inflammation and the bone phenotype observed in RA. The mechanistic insights derived from histopathological evaluation of joint tissues from patients with RA and experimental models of inflammatory arthritis show that TNFα suppresses bone repair through the overexpression of DKK1 and sclerostin [128, 129]. These mechanisms have significant implications for the development of strategies to prevent (by suppressing osteoclastogenesis and their recruitment) and/or revert bone erosions (by blockade of Wnt antagonists) in RA. Studies in animal models of RA indeed suggest that bisphosphonates reduce focal bone erosions and decrease juxtaarticular trabecular bone loss, but fail to control synovial inflammation [130]. Newer studies in RA patients suggest that the use of bisphosphonates in conjunction with anti-inflammatory therapy have a beneficial effect on bone mineral density (BMD) compared to patients only receiving anti-inflammatory treatment [131]. In alignment, a recent phase III study in RA patients receiving synthetic disease modifying anti-rheumatic drugs (DMARDs) and the anti-RANKL antibody denosumab found that denosumab reduced bone erosions and increased BMD compared to placebo [132]. In light of data from experimental models of arthritis showing that controlling bone destruction have limited effect on inflammation [127], while data in human suggest an added benefit of bisphosphonates when combined with anti-inflammatory treatment [131], it is likely that targeting bone destruction in humans may prevent tissue damage but pain and swelling will persist without some form of anti-inflammatory treatment to go along. Generalized bone loss involving trabecular and cortical bone in the axial and appendicular skeleton is also well documented in RA patients and is associated with an increased risk of hip and vertebral fractures [6, 117]. The factors responsible for generalized bone loss are rather difficult to define, primarily due to the multifactorial nature and the interplay between them. These factors include age, physical activity, systemic inflammation intensity and duration, and the use of glucocorticoids and other drugs, all of which independently effect bone remodeling [133, 134]. Interestingly, systemic anti-citrullinated protein antibodies (ACPA) - highly specific for RA - are also an inducer of osteoclastogenesis and can activate osteoclasts directly, thereby contributing to the local and generalized bone loss seen in RA [135]. These findings go in line with the clinical findings that ACPA in RA patients independently predicts higher risk of bone erosions and low systemic BMD [136–140]. It should be emphasized that a significant amount of generalized bone loss, correlated with levels of disease activity, appears early in RA [134, 141, 142]. Thus, assessment of magnitude of generalized bone loss, traditionally measured as BMD using dual energy X-ray absorption (DEXA) scans, is mostly relevant for early interventions in the “window of opportunity”.

### Tissue turnover markers in rheumatoid arthritis

The last decade has seen an increasing number of novel treatments targeting a variety of different immunological pathways involved in the RA pathology, including TNFα, IL-1 and IL-6 production and intracellular pathways such Janus kinase (JAK) inhibitors. Treatment effects are determined by the degree of disease activity, patient-reported quality of life, and acute phase responses, such as erythrocyte sedimentation rate (ESR) and C-reactive protein (CRP). While markers of inflammation can clinically be relevant for determining treatment response, markers that could accurately describe tissue damage in the different joint compartments would likely be better suitable for guiding targeted treatment.

Several serological markers which are based on measuring fragments released from the matrix in both bone and cartilage, as a result of increased tissue destruction or turnover, exist in the RA arena (Table 3). The Cathepsin K generated bone biomarkers CTX-I and N-terminal telopeptide (NTX) 1 are measures of osteoclast mediated bone resorption, as exemplified by the reduction of these markers in osteoporosis patients treated with anti-resorptive drugs [109]. In RA, different factors, such as systemic inflammation, glucocorticoid use, and menopause can affect bone turnover. Baseline CTX-I levels are only to some degree correlated to joint damage in RA, suggesting that Cathepsin K driven bone resorption may be less prevalent in RA [143, 153]. Indeed, increased levels of serum ICTP and C1M in RA compared to controls, and the association between C1M levels and radiographic progression suggest that osteoclasts may induce MMP-mediated matrix degradation in RA [144, 154, 155]. Also, several clinical studies have shown reductions in the levels of ICTP and C1M in response to infliximab, tocilizumab or the spleen tyrosine kinase (SYK) inhibitor fostamatinib, which suggests a more MMP driven pathology [144–146, 156]. The bone formation marker osteocalcin is lower in untreated RA patients compared to controls [148, 149], while anti-inflammatory treatment seems to normalize this.

**Table 3:** Biomarkers at the bone cartilage interface in RA

Biomarker	Disease Levels compared to healthy	Findings	References
**Bone turnover**			
CTX-I	Conflicting	Correlated with joint damage, radiographic progression and response to treatment	[143–146]
ICTP	Increased	Correlated with joint damage	[145, 146]
C1M	Increased	Correlate with joint damage (JSN, mtss) and radiographic progression	[147] [85]
Osteocalcin	Reduced (in naïve comparedto healthy controls)	Predictive of treatment response to anti-IL-6R therapy in combination withbiomarkers CTX-I and C2M	[144, 148, 149]
**Cartilage Turnover**			
CTX-II	Increased	Associated with radiographic progression	[150]
C2M	–	Low levels associated with anti-Il-6R treatment response by swollen andtender joint count in composite with CTX-I and osteocalcin	[144]
	–	–	
PIIANP	decreased	–	[151]
**Synovium turnover**			
C4M	Increased	Associated with anti-IL-6R treatment efficacy. Baseline levels associated withstructural progression by JSN and Sharp score	[152]

*mtss* Modified total Sharp score

Bone biomarkers have also shown promise in predicting response to treatment. A recent study found that RA patients who had a high bone turnover levels measured as the ratio between serum CTX-I and osteocalcin and who simultaneously had low serum C2M levels, indicating slow cartilage degradation, were more likely to respond to anti-IL-6R treatment [144].

Cartilage turnover markers have also proven useful in RA. High levels of CTX-II predict an increased risk of radiographic progression [150]. CTX-II is persistently increased in RA, while the formation marker PIIANP seems to decrease over time with increasing RA duration. These biomarker findings corroborate the notion that cartilage deterioration in RA is both driven by increased cartilage destruction and anti-anabolic effects limiting cartilage repair at the same time [151].

Type IV collagen is an abundant protein of basement membranes, but it is also part of the interstitial matrix of the synovial lining layer. The MMP-generated neo-epitope fragment of type IV collagen, C4M, was found to be associated with treatment efficacy in two phase III clinical studies with tocilizumab. Furthermore, higher baseline levels were associated with structural progression in the form of joint space narrowing (JSN) and Sharp score after 52 weeks. Tocilizumab treatment decreased C4M levels in a dose dependent manner and early reduction was associated with a better treatment response [152]. In summary, biomarkers for RA have shown to be useful for drug efficacy and disease activity. However, understanding and describing the endotype of the individual RA patient by biomarkers, may lead to better personalized medicine.

### Psoriatic arthritis – the local bone erosion and bone formation phenotype

PsA is a form of inflammatory arthritis associated with psoriasis [157]. A significant difference between PsA and other forms of inflammatory arthritis is an altered bone remodeling which manifests not only as increased bone resorption with bone erosions, osteolysis, and loss of bone mineral density, but also as increased bone formation such as syndesmophytes, enthesophytes and ankylosis (Fig. 3) [158]. In PsA, the normal bone remodeling homeostasis and communication between osteoclasts and osteoblasts is perturbed. An array of cytokines and growth factors associated with differentiation and function of osteoclasts and osteoblasts are altered, resulting in the different bone remodeling subtypes in PsA [159].

Although the bone resorptive phenotype in both RA and PsA appear similar, at least early in disease, there are significant differences between the two. RA typically involves phalangeal joints in a symmetric pattern with bone erosions and inadequate repair mechanisms resulting in local and systemic bone loss [160, 161]. PsA often exhibits a ray-like asymmetric pattern involving any of the peripheral joints. The bone erosions in PsA are often smaller with more ill-defined margins than those in RA because of periosteal new bone formation adjacent to erosions [162, 163], suggesting different pathological mechanisms underlying the bone changes in these diseases.

Both RANK and RANKL expressing cells have been shown to be highly elevated in PsA compared to OA and the upregulated RANKL expression was highly localized to the synovial lining layer [164]. Measuring levels of RANKL in serum of PsA patients has also revealed much higher levels of RANKL compared to both plaque psoriasis without arthritis and healthy subjects [165].

TNFα, like in RA, also plays a central role in the pathophysiology of PsA. The levels of TNFα and its soluble receptor (TNF-R55) are elevated in serum and synovial fluid of PsA patients [166]. Furthermore, treatment with anti-TNFα in PsA has demonstrated inhibition of radiographic progression and this seems to correlate to a reduction in circulating osteoclast precursors, indicating an osteoclast directed effect of TNFα [167]. Although most studies have found that the levels of TNFα and related cytokines are somewhat lower in PsA than those in RA, the role of TNFα in inflammation and joint destruction does not seem particularly different [168, 169].

IL-17 is another prominent cytokine that has been implicated in inflammatory joint diseases – including RA, PsA and AS. In both PsA and psoriasis patients, IL-17 secretion by T helper 17 (T_h_17 cells is significantly elevated compared to healthy controls [170]. IL-17 has been found to stimulate osteoclastogenesis by upregulating osteoblastic genes such as RANKL in co-culture models of osteoblasts and osteoclast-precursors [171]. IL-17 can also promote osteoclastogenesis from osteoclast precursors in the absence of osteoblasts and RANKL – an effect which could be interrupted by treatment with the TNFα inhibitor infliximab, indicating that this differentiation may be associated with TNFα signaling [172]. Furthermore, IL-17A gene transfer to C57BL/6 J mice in a collagen-induced arthritis (CIA) model of inflammatory arthritis showed elevated levels of IL-17RA^+^CD11b^+^Gr1^low^RANK^+^CSF-1R^+^ osteoclast precursors with increased differentiation capacity compared to precursors from control mice even before arthritis onset, suggesting that IL-17A expression may exacerbate bone destruction even in the absence of inflammation [173]. In line with this, recent clinical studies have demonstrated efficacy in reducing arthritis and structural damage in PsA patients treated with secukinumab, an IL-17 inhibiting antibody [174].

IL-23 is involved in bone remodeling by inducing osteoclastogenesis but also modulates differentiation of IL-17 and IL-22 secreting immune cells. The secretion of IL-17, − 22 and − 23 has also been associated with arthritis and enthesitis in a mouse model of SpA [175]. Perhaps more convincing evidence for the involvement of IL-23 in bone destruction is the effect of a p40 subunit (of IL-23) targeting antibody, in limiting structural progression in phase III clinical trials [176], as well as recent findings from phase III clinical trials on the IL-23 specific subunit p19 antibody Guselkumab showing significant reduction in radiographic score [177].

While the mechanisms underlying bone destruction in PsA are relatively well described, the mechanisms underlying the bone formation typically observed in PsA are still not well understood. The excessive bone formation can occur both in the axial skeleton, presenting as syndesmophytes, and in the peripheral joints, as joint ankylosis, enthesophytes or periosteal bone formation [178]. Although appearing similar, bone formation in PsA differs somewhat from the pathology observed in AS. The main difference is an often asymmetric pattern of bone formation associated with PsA, with syndesmophytes being distributed and randomly progressing along the spine, in contrast to the more symmetrical manifestation observed in AS patients.

The bone formation in PsA arises from anabolic factors that stimulate the formation of hypertrophic chondrocytes and osteoblasts. However, the link between inflammatory cytokines and this bone formation is still elusive. Nonetheless, the importance of Wnt/β-catenin signaling in directing osteoblast differentiation and function in PsA is well established. In contrast to RA, decreased serum levels of Wnt pathway antagonist DKK1 have been reported in PsA patients as compared to healthy controls which is in line with an increase in bone formation [179]. Moreover, serum levels of DKK1 were shown to be inversely correlated to development of syndesomophytes in AS patients [180]. However, reports also show increased DKK1 levels in PsA, which highlights the complex bone phenotype involving both erosion of bone and aberrant bone formation [181, 182].

Evidence of the involvement of BMP signaling has been suggested by studies in DBA/1 mice that spontaneously develop PsA-like arthritis, but also ankylosing enthesitis [183, 184]. These mice exhibited elevated expression of BMP and increased SMAD 1/5 phosphorylation in the ankylosing entheses. Overexpression of the BMP antagonist Noggin led to a reduction in joint pathology [184]. In addition, serum BMP-7 was correlated to enthesitis in the male mice [183]. These findings implicate that BMP signaling is associated with bone formation in PsA.

### Tissue turnover markers in psoriatic arthritis

Assessment of biomarkers reflecting bone turnover in human PsA patients has shown variable results depending on the stage and activity of the disease (Table 4). There are conflicting results on levels of bone resorption markers, like CTX-I and ICTP, as compared to healthy controls [179, 185, 186]. On the contrary, C1M is found to be significantly increased in PsA [186].

**Table 4:** Biomarkers at the bone cartilage interface in PsA

Biomarker	Disease Levels compared to healthy	Findings	References
**Bone turnover**			
CTX-I	Conflicting reports	–	[179, 185]
ICTP	Conflicting reports	–	[179, 185, 186]
Osteocalcin	Increased, but conflicting reports	–	[187, 188] [182]
C1M	Increased	–	[186]
BALP	Increased, but conflicting reports	–	[187, 188] [182]
**Cartilage Turnover**			
CTX-II	Increased	–	[189]
C2M	No difference	–	[190]
C2C	Increased	–	[191]
PIIANP	Increased	–	[192]
PIIBNP	Increased	–	[193]
CPII	Increased	–	[191]
COMP	Increased in synovial fluid compared to RA	No clear correlation with disease activity	[191, 194–196]

In contrast to RA, increased levels of bone formation markers, osteocalcin and bone specific ALP, have been reported for PsA patients in some studies [187, 188] but these findings are not consistent [182] and data on their correlation with radiographic progression in PsA is still missing. Other mediators of bone remodeling such as RANKL, M-CSF and DKK1 along with MMP-3 have outperformed the traditional bone turnover biomarkers [179, 181, 197, 198]. A growing body of evidence suggests that these markers have diagnostic value, but associations with radiographic progression are still controversial. Serum MMP-3 levels can possibly predict clinical response to anti-TNFα therapy [199–201].

Both the cartilage formation markers PRO-C2 [193], CPII [191] and PIIANP [190], and the cartilage degradation markers CTX-II [189], and C2C [191], have been shown to be elevated in the serum of PsA patients [190, 191], suggesting increased turnover of collagen type II. However, C2M (a degradation marker) levels were similar to those in healthy controls [190]. COMP, another cartilage biomarker, is indicated to be elevated in serum and synovial fluid of PsA patients. It has been shown that COMP levels are higher in synovial fluid of PsA patients as compared to RA patients [194], but results concerning correlation of COMP and PsA disease activity are inconclusive [191, 195, 196].

Together with bone and cartilage biomarkers in PsA, skin biomarkers may be useful tools to identify the psoriatic component of PsA. For psoriasis the ideal biomarker has not yet been identified, however serum measurements of different S100 proteins are associated with disease activity [202, 203], VEGF [204, 205] and IL-18 [206] has been suggested as biomarkers of disease severity, while CRP [207] and TNFα [206] are general inflammatory biomarkers associated with skin disease. In summary, changes in both cartilage, bone and skin are associated with PsA, and its underlying pathology. Therefore, treatments for PsA may need to target the disease pathway, and a panel of biomarkers reflecting the different tissues are needed to provide an accurate profile of the patient and identify the individual patients endotype.

### Ankylosing spondyloarthritis – the local endochondral bone formation and fibrosis phenotype

AS is part of a group of diseases termed spondyloarthritides, characterized by chronic inflammation affecting the spine and sacroiliac joints. It typically presents in young adults and imposes a significant burden. AS is diagnosed by symptom, a physical exam, inflammation markers and by radiography or MRI. AS ss characterized by the presence of structural changes to the skeleton, mainly manifesting as progressive ankylosis of the spine and the sacroiliac joints, but also by inflammation associated trabecular bone loss resulting in increased fracture risk (Fig. 3). Both types of bone pathology contribute to loss of function and disability. In contrast to both RA and PsA, involvement of peripheral joints usually is limited.

The bone phenotype in AS is a complex mix of different processes where both pathological bone loss and excessive bone formation shape the clinical manifestation of the disease [208]. The loss of bone may be systemic or present as local bone erosions in the sacroiliac joints and vertebrae – although bone erosions are more common for other forms of SpA. The bone loss in AS may be partly explained by mechanisms as those seen in RA. In line with this, and similar to RA, bisphosphonates have been investigated with the purpose to evaluate how it affects bone turnover in SpA. Current trials have shown that bisphosphonates decrease disease activity, bone erosions and subsequently reduce bone formation [209–211]. Specifically, the amino-biphosphonate neridronate has been compared head-to-head with infliximab, the anti-TNFα inhibitor, where they were equally effective in reducing disease activity in AS, together with an improvement on BMD changes [211].

The mechanisms responsible for the characteristic alterations to bone formation leading to ankylosis between adjacent vertebrae or other bones is still a feature of the disease which is not fully understood. It is proposed that the process is initiated around entheses, the connection or transition zone between tendons or ligaments and bone [212, 213]. The process of ankylosing enthesitis appears to involve signaling between BMPs and Wnts and cells involved in bone remodeling and bone formation. How inflammation is associated with activation and regulation of these factors and pathways is still under investigation. Pre-clinical animal models suggest that persistent inflammation in the subchondral bone marrow is a pre-requisite for osteoproliferation that eventually leads to vertebral body deformity and fusion [214]. In humans, the proposed link between inflammation and new bone formation is supported by the finding that early anti-inflammatory treatment shows efficacy on progression of bone formation later in disease [215, 216].

Polymorphisms in BMP6 have been associated with radiologic severity in Korean AS patients [217]. However, serum concentrations of BMPs in AS patients have yielded differing results, where some studies have found increased BMP levels [218, 219], while others have reported no significant increase [220]. One study found an imbalance between Noggin and BMP2 which could cause abnormal osteogenic differentiation of mesenchymal stem cells, and indicates excessive osteoblast differentiation as a mechanism behind the pathological bone formation in AS [221]. Activation of BMP-2 and 6 was also found in synovial tissue of RA and AS patients [222], which could mean that the differing bone phenotypes between the two diseases results from the anatomical site of inflammation rather than the mechanism itself (entheses vs joint) [7].

Like in PsA, Wnt signaling has been implicated in the bone formation associated with AS. Preclinical evidence comes from studies in a human TNFα transgenic mouse model where inhibition of the wnt signaling antagonist DKK1 resulted in a switch from RA-like erosive phenotype to bone formation phenotype with progressive ankylosis of both limb and sacroiliac joints, suggesting that Wnts may promote bone formation associated with pathological development of AS [128, 223]. Although reduced levels of serum DKK1 have been reported in animal models of AS [224], increased levels of serum DKK1 as compared to healthy controls have consistently been reported in AS patients [225, 226]. Despite increased circulating levels, functionality of DKK1 (as measured by its binding to low-density lipoprotein receptor-related protein 6 (LRP6) receptor) is significantly decreased in AS patients [128, 225], which is in sharp contrast to the increased serum levels concomitant with enhanced functionality in RA patients [128]. Neither total serum nor functional DKK1 levels correlate with clinical disease scores in AS [128, 225], but a study by Heiland et al. [180] found a correlation between low functional levels of DKK1 and a more severe radiographic phenotype in AS. Inflammation-intensity dependent activation of Wnt signaling concomitant with ectopic new bone formation in spinal tissue from AS patients [227] and elevated serum levels of Wnt proteins in AS patients [227, 228], further support a role of Wnt signaling in AS. Interestingly, elevated serum Wnt3a levels were associated with clinical and radiographic progression, and could be a promising biomarker for osteoproliferative changes in AS [228].

### Tissue turnover markers in ankylosing spondylitis

Several studies evaluated the serum levels of bone turnover markers in AS, with inconsistent results. One explanation for this, could be the challenge of relating changes occurring in a single joint or enthesis, and compare it too systemic levels of low systemic inflammation. The systemic measurement may therefore disturb the serological measurement in terms of bone formation. In contrast to the observed pathological changes, most studies found no difference in serum levels of bone formation markers, like osteocalcin, PINP and bone specific ALP, in patients with AS compared with their control groups [226, 229–232]. However, increased osteocalcin [230] and PINP levels [233] in AS patients, if present, correlate well with the progression of radiographic syndesmophytes and ankylosis (Table 5).

**Table 5:** Biomarkers at the bone cartilage interface in AS

Biomarker	Disease Levels compared to healthy	Findings	References
**Bone turnover**			
CTX-I	Increased	Associated with radiographic progression No effect of anti-TNFα therapy	[229, 230, 233, 234]
ICTP	Increased	–	[235]
PINP	No difference	Correlated with radiographic progression of syndesmophytes and ankylosis	[226, 229–233]
Osteocalcin	No or limited difference	Associated with syndesmophytes and ankylosis	[226, 229–232]
BALP	No or limited difference	–	[226, 229–233]
**Cartilage Turnover**			
CTX-II	Increased	Correlated with change in clinical disease severity in response to anti-TNFα therapy	[229, 236]
C2M	Increased	–	[237]
C2C	No difference	–	[238]
PIIANP	Increased	–	[192]
PIIBNP	Increased	–	[193]

CTX-I is elevated in the serum [230] and urine [229] of AS patients, and is associated with radiographic progression [233]. However, serum CTX-I levels remain unaltered after anti-TNFα treatment in these patients [234]. Other biomarkers of bone resorption, like ICTP [235] have also been suggested of clinical value.

The serological patterns of cartilage turnover biomarkers in AS are very similar to PsA, whereby both formation markers, like PIIANP [192], CPII [238] and PRO-C2 [193], and degradation markers, like urinary CTX-II levels [229] are elevated. Moreover, urinary CTX-II is reduced in response to anti-TNF α therapy and the change correlates well with clinical severity [236]. The distinguishing feature is C2M which is elevated in AS patients but not in PsA [239] and C2C which is elevated in PsA but not in AS [238]. However, since AS is including a more fibrotic process than PsA, biomarkers including bone, cartilage and fibrosis markers may reflect the AS endotype more accurately.

### Defining common denominators across disease indications – common endotypes?

The bone manifestations listed here, while still arising from different mechanism and arising in different tissue areas, share some degree of similarity in their manifestation; namely the aberrant remodelling leading to alterations in bone density and architecture, and the osteochondral ossification like alterations leading to undesired calcification and bone growth or pannus formation. We hypothesize that endotypes across joint diseases exist which share similarities that can be exploited both when trying to treat the right patient with the right drug or potentially when repurposing drugs from other indications. The challenge of course lies in defining such endotype. Efforts are gaining traction in the OA field, where a number of endotypes have been suggested, among these, a bone driven endotype. For other rheumatic diseases involving bone alterations such as RA, the focus has rather been in trying to understand the underlying inflammatory patterns in order to better target anti-inflammatory treatment, and less attention have been given to structural aspects. Specific and sensitive biomarkers such as imaging and biochemical markers, that can accurately describe structural changes, combined with clinical assessment, seems crucial to defining common underlying endotypes. Longitudinal clinical studies including early to late stage disease patients with careful clinical examinations and state of the art imaging combined with continuous and consistent body fluid sampling would further enable us to study the link between systemic biomarkers and disease specific pathological bone turnover.

## Conclusion

It is apparent that unique molecular pathways underlie the manifestation of typical bone phenotypes in rheumatic disease. Common to all rheumatic diseases is aberrant bone homeostasis, leading to both excessive destruction and deposition of bone matrix in bones, joints and the spine, causing pain and disability. Despite the differing nature of the molecular processes in bone between different rheumatic diseases, biomarker signatures and platforms provide a valuable opportunity to objectively define the underlying pathobiology, which largely recapitulate the phenotypic changes arising with disease onset, progression and treatment. With the varying phenotypes, underlying aetiologies, and in some rheumatic diseases a lack of effective responses to treatment in bone, joint and spine disease, the field is becoming increasingly focused on application of biomarkers to accurately identify patient profiles and ensure more appropriate and targeted treatment, ultimately working towards more personalized healthcare approaches. In the coming years, it will be increasingly important to test markers in both well characterized, longitudinal observational cohorts to better understand the natural courses of the diseases and intervention studies in order to identify which patients are more likely to respond to treatment, for the benefit of patients, healthcare systems, and pharmaceutical companies.

## Data Availability

Not applicable.

## Abbreviations

*ACVR1*: Activin A receptor type I
ACPA: Anti-citrullinated peptide antibody
ADOII: Autosomal dominant osteopetrosis type II
ALK-2: Activin receptor-like kinase-2
AS: Ankylosing spondylitis
BMD: Bone mineral density
BML: Bone marrow lesion
BMP: Bone morphogenic protein
(B)ALP: (Bone specific) alkaline phosphatase
CIA: Collagen induced arthritis
COMP: Cartilage oligomeric protein
CRP: C-reactive protein
CTX: C-terminal telopeptide
DEXA: Dual-energy X-ray absorptiometry
ECM: Extracellular matrix
DKK1: Dickkopf-related protein 1
DMARD: Disease modifying rheumatoid arthritis drug
DMOAD: Disease modifying osteoarthritis drug
ESR: Erythrocyte sedimentation rate
FLS: Fibroblast like synoviocyte
FOP: Fibrodysplasia ossificans progressive
ICTP: Collagen type I C-terminal telopeptide
IL: Interleukin
JAK: Janus kinase
JSN: Joint space narrowing
KL: Kellgren-Lawrence
LRP6: Low-density lipoprotein receptor-related protein 6
M-CSF: Macrophage colony-stimulating factor
MMP: Matrix-metalloproteinase
MRI: Magnetic resonance imaging
mtss: Modified total Sharp score
NTX: N-terminal telopeptide
OA: Osteoarthritis
OPG: Osteprotegerin
OPT: Osteopetrosis
OP: Osteoporosis
PINP: Amino-terminal propeptide of type 1 procollagen
PsA: Psoriatic arthritis
RANK(L): Receptor activator of nuclear factor kappa-Β (ligand)
RA: Rheumatoid arthritis
SFRP-1: Secreted frizzled-related protein 1
SOST: Sclerostin
SYK: Spleen tyrosine kinase
TRACP: Tartrate-resistant acid phosphatase
TGFβ: Transforming growth factor β
T_h_17: T-helper 17
TNFα: Tumor necrosis factor α
VEGF: Vascular epithelial growth factor
